# Supplementary material for: Multidisciplinary Care of Patients with Facial Palsy: Treatment of 1220 Patients in a German Facial Nerve Center
Source: J Clin Med. 2022 Jan 14;11(2):427. doi: 10.3390/jcm11020427 (PMC8778429; doi:10.3390/jcm11020427)
Supplement: Supplementary file 1 [file jcm-11-00427-s001.zip › jcm-1509047 supplementary.pdf]

SUPPLEMENT TABLES AND SUPPLEMENT FIGURESA
Multidisciplinary care of patients with facial palsy: Treatment of 1220 patients in a German facial nerve center

Jonathan Steinhäuser[1], Gerd Fabian Volk[1,2,3], Jovanna Thielker[1,2], Maren Geitner[1,2], A.-M. Kuttenreich[1,2], Carsten M. Klingner[2,3,4], Christian Dobel[1,2], and Orlando Guntinas-Lichius, MD[1,2,3]*

[1]Department of Otorhinolaryngology, Jena University Hospital, Jena, Germany
[2]Facial Nerve Center Jena, Jena University Hospital, Jena, Germany
[3]Center for Rare Diseases Jena University Hospital, Jena, Germany
[3]Department of Neurology, Jena University Hospital, Jena, Germany

Supplement Tables

Supplement Table S1

**Supplement Table S1.** Patients' characteristics (N=1220)		
**Parameter**	**Absolute (N)**	**Relative (%)**
Gender		
Female	713	58.4
Male	507	41.6
Side		
Right	567	46.5
Left	645	52.9
Bilateral	8	0.7
Recurrent palsy		
No	1146	93.9
Yes	74	6.1
Localization		
peripheral	1211	99.3
central	8	0.7
nuclear	1	0.1
Etiology		
idiopathic	574	47.0
thereof classical Bell's palsy	496	40.7
thereof pregnancy	22	1.8
thereof recurrent	56	4.6
iatrogenic	234	19.2
infectious/inflammatory	198	16.2
thereof varicella zoster	128	10.5
thereof borreliosis	35	2.9
thereof autoimmune	17	1.4
thereof otogenic	9	0.7
thereof other	9	0.7
traumatic	42	3.5
thereof temporal bone trauma	27	2.2
thereof other trauma	15	1.2
neoplastic	147	12.0
thereof vestibular schwannoma postop/postRT*	102	8.4
thereof malignant tumor	28	2.3
thereof benign tumor	17	1.4
congenital	25	2.0
	**Mean±SD**	**Median, range**
Age (years) at initial diagnosis	48.0±20.2	50, 0-91

Age (years) at initial presentation	50.2±18.7	52, 0-91
Interval (years) onset to initial presentation	2.3±6.6	0.02, 0-71
Interval (years) onset to initial presentation, acute palsy	0.02±0.04	0.003, 0-0.25
Interval (years) onset to initial presentation, chronic palsy	5.3±9.2	1.5, 0.2-70
Interval (years) onset to last presentation	3.4±7.0	1.0, 0-71
Interval (years) onset to last presentation, acute palsy	0.9±1.9	0.9, 0-14
Interval (years) onset to last presentation, chronic palsy	6.7±9.4	3.0, 0.2-70
Interval (years) first to last presentation	1.1±1.9	0.4, 0-14
Interval (years) first to last presentation, acute palsy	0.9±1.9	0.3, 0-14
Interval (years) first to last presentation, chronic palsy	1.4±1.9	0.9, 0-13

SD = standard deviation; RT = radiotherapy

Supplement Table S2

**Supplement Table 2.** Overview about absolute number of diagnostics (N=1220) and frequency per patient.		
**Parameter**	**Absolute (N)**	**Relative (%)**
Sonography of the neck		
Yes	505	41.4
No	715	58.6
Sonography of the facial muscles		
Yes	193	15.8
No	1027	84.2
Magnet resonance imaging, cranial		
Yes	137	11.2
No	1083	88.8
Computed tomography, cranial		
Yes	88	7.2
No	1132	92.8
Facial electrophysiology		
Yes	1066	87.4
No	154	12.6
Facial photo series		
Yes	1116	91.5
No	104	8.5
Audiometry		
Yes	843	69.1
No	377	30.9
Tympanometry		
Yes	807	66.1
No	413	339
Stapedius reflex test		
Yes	789	64.7
No	431	35.3
Gustatory test		
Yes	733	60.1
No	487	39.9
Vestibular tests		
Yes	696	57.0
No	524	43.0
Schirmer test		
Yes	681	55.8
No	539	44.2
Serology		
Yes	441	36.1
No	779	63.9
	**Mean±SD**	**Median, range**
Sonography of the neck	0.6±0.9	1, 0-15
Sonography of the facial muscles	0.6±1.8	0, 0-16
Magnet resonance imaging, cranial	0.1±0.4	0, 0-6
Computed tomography, cranial	0.1±0.3	0, 0-4
Facial electrophysiology	1.6±2.0	1, 0-20
Audiometry	0.8±0.8	1, 0-7
Tympanometry	0.7±0.8	1, 0-4
Stapedius reflex test	0.7±0.6	1, 0-3
Gustatory test	0.6±0.6	0, 0-3
Vestibular tests	0.6±0.6	1, 0-6
Schirmer test	0.6±0.5	1, 0-3
Serology	0.4±0.6	0, 0-3

SD = standard deviation.

Supplement Table S3

**Supplement Table 3.** Treatment of all patients (N=1220).					
**Parameter**	**Absolute**	**Relative**	**Time from onset of facial palsy to treatment in Facial Nerve Center, days**		
	**(N)**	**(%)**	**Mean±SD**	**Median**	**Range**
Drug treatment					
Glucocorticoids	571	46.8	2.4±7.3	1	0-132
Glucocorticoids, because of acute facial palsy	564	46.2	2.2±4.9	1	0-57
Glucocorticoids, because of acute Bell's palsy	322	26.3	2.8±5.3	1	0.57
Acyclovir (all because of acute Bell's palsy)	386	31.6	2.8±5.5	1	0-62
Antibiotics	63	5.2	7.4±22.4	2	0-153
Antibiotics, because of acute facial palsy	60	4.9	6.1±19.9	2	0-153
Antibiotics, because of acute borreliosis	20	1.6	3.3±4.4	2	0-17
Facial nerve reconstruction					
Facial-facial nerve suture	4	0.3	55.7±71.2	29	0-174
Facial nerve interpositional graft	8	0.7	328.8±585	49	0-1625
Hypoglossal-facial-nerve jump suture	41	3.4	1354.9±2102.9	480	0-9238
Muscle and sling plasty					
Temporal muscle transfer	1	0.1	659	659	659
Sling plasty angle of the mouth	16	1.3	2320.0±5414.2	627	52-21937
Eye lid surgery					
Upper eye lid weight	77	6.2	1345.7±2940.3	377	0-21937
Tarsorrhaphy	10	0.8	983.9±830.2	718	3-2615
Kanthopexy	16	1.3	1317.9±1676.9	621	52-5734
Brow plasty	22	1.8	1557.0±1767.2	671	52-6549
Blepharoplasty	15	1.2	3357.5±5087.9	988	218-18943
Lower lid plasty	25	2.0	1050.0±1200.5	538	48-4618
Non-surgical adjuvant therapy					
Physical therapy/speech therapy	102	8.3	1096.7±2234.1	521	1-17259
Electrotherapy	58	4.7	2172.9±3608.6	669	21-22355
Botulinumtoxin injection	173	14.0	1756.6±2835.4	655	19-17456
Eye moisture chamber	435	35.3	51.1±396.1	1	0-5547
Eye drops/ointment	450	36.5	117.6±995.6	1	0-16719
Facial exercises at home	455	36.9	44.5±40.63	1	0-7352
Facial EMG biofeedback training	286	28.6	1992.9±2857.2	872	124-23369

SD = standard deviation.

Supplement Table S4

**Supplement Table S4.** Facial nerve function at initial and last visit (N=1220)		
**Parameter**	**Absolute (N)**	**Relative (%)**
Facial function of disease at first visit		
Acute phase (≤90 days after onset)	697	57.1
thereof incomplete	577	47.3
thereof complete	119	9.8
thereof complete recovery	1	0.1
Chronic phase (>90 days after onset)	523	42.8
thereof incomplete	99	8.1
thereof complete	90	7.4
thereof postparalytic synkinesis	330	27.0
thereof complete recovery	4	0.3
Facial function at last visit		
Acute phase (≤90 days after onset)	329	30.0
thereof incomplete	67	6.0
thereof complete	11	0.9
thereof postparalytic synkinesis	1	0.1
thereof complete recovery	250	20.5
Chronic phase (>90 days after onset)	891	73.0
thereof incomplete	136	11.0
thereof complete	80	6.6
thereof postparalytic synkinesis	459	37.6
thereof complete recovery	216	17.7
	**Mean±SD**	**Median, range**
Interval (years) first to last presentation	1.1±1.9	0.4, 0-14.1
Number of visits	2.6±3.6	1, 1-52

*N = 1073 patients with at least two visits; SD = standard deviation.

Supplement Table S5

**Supplement Table S5.** Comparison of outcome measures between first and last visit.*									
	**All patients N=1220**			**Acute palsy N=697**			**Chronic palsy N=523**		
	**First visit Mean±SD**	**Last visit Mean±SD**	**p****	**First visit Mean±SD**	**Last visit Mean±SD**	**p****	**First visit Mean±SD**	**Last visit Mean±SD**	**p****
Stennert index, sum score	4.8±3.0	2.1±2.7	**<0.0001**	5.1±2.9	1.2±2.3	**<0.0001**	4.2±3.0	3.6±2.7	**<0.0001**
FDI, physical subscore	62.8±19.0	75.8±17.4	**<0.0001**	62.0±19.5	80.0±18.2	**<0.0001**	63.1±18.8	73.3±16.5	**<0.0001**
FDI, social subscore	67.6±19.9	76.2±17.5	**<0.0001**	73.1±17.5	80.2±16.1	**<0.0001**	64.4±20.5	73.8±17.8	**<0.0001**
FDI, total score	64.9±17.1	77.8±41.2	**<0.0001**	67.0±16.9	85.6±64.3	**<0.0001**	63.6±17.1	73.3±15.9	**<0.0001**
FaCE Facial movement	36.5±24.5	50.8±27.2	**<0.0001**	40.7±27.5	66.7±30.3	**<0.0001**	34.1±22.5	42.1±20.8	**<0.0001**
FaCE Facial comfort	51.8±29.6	64.8±28.4	**<0.0001**	61.5±27.3	75.3±26.6	**<0.0001**	46.5±29.7	59.2±27.8	**<0.0001**
FaCE Oral function	67.5±27.6	80.1±24.1	**<0.0001**	69.5±25.4	86.7±22.7	**<0.0001**	66.5±28.7	76.3±24.1	**<0.0001**
FaCE Eye comfort	46.2±33.4	60.1±33.2	**<0.0001**	42.9±31.9	69.9±32.3	**<0.0001**	48.4±34.1	54.9±32.5	**<0.0001**
FaCE Lacrimal control	57.2±34.3	67.7±30.4	**<0.0001**	51.8±35.5	75.0±29.9	**<0.0001**	60.4±33.2	64.0±30.0	**0.043**
FaCE Social function	66.2±26.6	80.8±23.3	**<0.0001**	73.9±25.3	90.2±19.3	**<0.0001**	62.3±26.3	75.7±23.7	**<0.0001**
FaCE Total score	54.6±19.9	67.5±20.1	**<0.0001**	58.1±20.8	77.2±21.1	**<0.0001**	52.7±19.1	62.8±17.8	**<0.0001**
SF-36 Physical functioning	81.8±22.2	84.0±22.5	**0.025**	81.9±21.2	84.5±22.2	**0.024**	79.9±26.5	81.2±26.0	0.281
SF-36 Role physical	65.6±42.0	74.8±38.1	**<0.0001**	63.2±42.5	77.3±37.5	**<0.0001**	70.2±41.1	71.3±39.0	0.002
SF-36 Bodily pain	72.7±27.0	80.0±24.7	**0.001**	70.1±28.2	82.4±23.1	**0.002**	76.0±24.9	75.1±27.2	0.106
SF-36 General health	59.2±18.9	63.5±20.8	**<0.0001**	59.3±16.2	65.2±21.4	**0.039**	58.4±22.7	60.2±20.0	**0.002**
SF-36 Vitality	55.8±18.3	59.9±17.2	**<0.0001**	58.1±18.3	62.3±17.5	**0.004**	51.0±19.0	55.0±17.6	**<0.0001**
SF-36 Social functioning	78.6±24.6	86.2±19.6	**<0.0001**	79.7±25.2	89.6±16.1	**<0.0001**	76.3±23.5	80.0±23.5	**<0.0001**
SF-36 Role emotional	75.5±38.2	84.5±32.8	**<0.0001**	77.8±37.7	88.5±30.4	**0.011**	72.7±38.9	78.6±35.5	**<0.0001**
SF-36 Mental health	67.0±20.0	73.3±17.6	**<0.0001**	68.6±20.7	74.9±17.4	**0.034**	64.4±18.3	70.5±17.8	**<0.0001**

N = 1073 patients with at least two visits; **significant p-values (p<0.05) in bold; FDI = Facial Disability Index; FaCE = Facial Clinimetric Evaluation Scale; SF-36 = 36-Item Short Form Survey; NA = not applicable; SD = standard deviation.

Supplement Table S6

**Supplement Table S6.** Comparison of outcome measures between first and last visit.*									
	**Glucocorticoids alone N=190**			**Glucocorticoids and acyclovir N=381**			**Botulinum toxin injections N=160**		
	**First visit Mean±SD**	**Last visit Mean±SD**	**p****	**First visit Mean±SD**	**Last visit Mean±SD**	**p****	**First visit Mean±SD**	**Last visit Mean±SD**	**p****
Stennert index, sum score	4.9±3.0	1.3±2.3	**<0.0001**	5.7±2.5	1.0±2.0	**<0.0001**	3.7±2.9	2.3±1.9	**<0.0001**
FDI, physical subscore	63.1±19.0	81.0±19.1	**<0.0001**	63.7±20.0	81.3±17.5	**<0.0001**	60.2±19.0	71.2±16.2	**<0.0001**
FDI, social subscore	71.5±20.1	78.9±17.4	**0.009**	75.9±15.2	81.3±16.3	**0.005**	61.6±18.5	74.1±16.6	**<0.0001**
FDI, total score	67.3±17.7	79.9±16.6	**<0.0001**	68.8±16.5	81.3±15.0	**<0.0001**	60.6±15.9	72.7±14.2	**<0.0001**
FaCE Facial movement	40.6±29.6	66.4±33.8	**<0.0001**	46.5±25.1	74.3±25.9	**<0.0001**	37.5±24.2	45.8±19.5	**<0.0001**
FaCE Facial comfort	56.8±30.1	69.7±31.7	**0.002**	66.1±26.9	82.9±23.2	**<0.0001**	42.6±30.5	51.4±26.7	**0.007**
FaCE Oral function	67.6±29.5	82.8±28.6	**0.003**	70.0±25.0	91.3±18.6	**<0.0001**	64.8±28.7	75.8±21.2	**<0.0001**
FaCE Eye comfort	48.7±35.7	68.9±35.6	**<0.0001**	43.1±30.1	76.5±27.4	**<0.0001**	45.9±32.2	54.4±30.3	**0.001**
FaCE Lacrimal control	53.6±37.9	78.6±32.2	**0.001**	48.7±33.2	74.3±29.0	**<0.0001**	61.0±32.7	65.2±29.3	0.213
FaCE Social function	71.4±27.1	86.6±24.5	**0.002**	78.7±23.0	94.6±14.0	**<0.0001**	58.5±25.8	75.6±23.2	**<0.0001**
FaCE Total score	56.5±25.1	74.5±24.4	**<0.0001**	62.8±19.4	83.8±16.9	**<0.0001**	50.5±19.7	61.1±17.1	**<0.0001**
SF-36 Physical functioning	81.3±23.0	81.3±22.9	0.724	81.4±21.6	85.1±23.5	0.144	76.1±27.5	71.5±32.9	0.874
SF-36 Role physical	66.6±47.3	78.1±40.7	0.151	61.0±42.1	76.7±36.3	**<0.0001**	62.5±39.2	60.4±47.0	**0.011**
SF-36 Bodily pain	69.6±25.0	76.8±25.3	0.260	70.0±28.6	83.9±23.6	**0.033**	70.0±29.0	64.8±24.5	0.334
SF-36 General health	59.3±15.4	63.2±17.1	0.276	58.8±17.3	65.6±23.4	**0.047**	53.7±20.2	62.1±23.4	0.142
SF-36 Vitality	54.6±13.1	64.3±18.2	0.541	61.0±20.1	62.7±17.0	**0.034**	50.4±20.2	52.7±18.7	**<0.0001**
SF-36 Social functioning	72.7±27.5	84.4±26.0	**0.026**	83.5±23.3	91.8±14.0	**0.004**	79.2±19.5	81.3±15.5	**0.002**
SF-36 Role emotional	64.3±46.2	76.2±42.2	0.943	81.6±34.8	91.5±26.3	**0.012**	71.8±40.5	76.9±39.4	**0.005**
SF-36 Mental health	63.5±18.5	74.1±16.6	0.292	72.5±19.6	75.9±17.7	0.059	66.2±19.4	75.4±19.2	**<0.0001**

*Out of N = 1073 patients with at least two visits; **significant p-values (p<0.05) in bold; FDI = Facial Disability Index; FaCE = Facial Clinimetric Evaluation Scale; SF-36 = 36-Item Short Form Survey; NA = not applicable; SD = standard deviation.

Supplement Table S7

**Supplement Table S7.** Comparison of outcome measures between first and last visit.*									
	**Hypoglossal-facial jump surgery N=41**			**Eye lid surgery N=92**			**EMG Biofeedback training N=285**		
	**First visit Mean±SD**	**Last visit Mean±SD**	**p****	**First visit Mean±SD**	**Last visit Mean±SD**	**p****	**First visit Mean±SD**	**Last visit Mean±SD**	**p****
Stennert index, sum score	8.7±1.3	6.8±2.5	**<0.0001**	7.2±2.9	6.1±3.1	**<0.0001**	3.8±2.8	2.8±2.0	**<0.0001**
FDI, physical subscore	63.4±16.1	71.6±16.0	**0.002**	58.6±17.0	65.0±14.6	**0.005**	61.8±18.3	73.8±18.3	**<0.0001**
FDI, social subscore	68.8±20.3	74.6±15.4	0.067	65.5±21.7	66.7±22.6	0.483	64.8±19.6	75.4±16.5	**<0.0001**
FDI, total score	66.2±16.2	73.3±14.4	**0.008**	61.6±17.1	65.8±16.7	**0.047**	63.2±16.5	74.7±14.4	**<0.0001**
FaCE Facial movement	20.0±14.9	25.4±20.3	0.053	25.3±17.1	27.2±20.0	0.273	34.9±21.8	44.8±18.6	**<0.0001**
FaCE Facial comfort	65.5±23.6	70.2±21.2	0.212	51.2±28.0	55.6±27.6	0.112	44.5±28.7	58.5±26.9	**<0.0001**
FaCE Oral function	69.6±27.5	74.1±26.1	0.382	60.7±31.3	63.1±29.6	0.531	66.7±26.7	78.4±21.2	**<0.0001**
FaCE Eye comfort	40.7±34.6	42.6±32.8	0.330	34.1±27.9	34.7±28.4	0.317	46.2±33.0	56.9±30.8	**<0.0001**
FaCE Lacrimal control	45.2±33.2	44.2±31.9	1.000	46.3±31.8	48.8±32.0	0.485	60.4±31.9	65.0±28.3	**0.025**
FaCE Social function	62.9±32.0	74.5±23.1	**0.008**	59.7±29.3	66.4±29.4	0.061	62.2±25.3	78.4±21.4	**<0.0001**
FaCE Total score	54.0±18.5	59.3±18.2	**0.005**	46.9±18.1	50.7±21.1	**0.015**	52.0±17.9	63.9±16.6	**<0.0001**
SF-36 Physical functioning	79.4±20.4	85.8±9.7	**0.015**	77.2±24.4	73.5±27.2	0.223	81.7±25.8	86.0±19.2	0.585
SF-36 Role physical	79.2±33.2	83.3±20.4	0.051	66.7±50.0	58.3±46.1	0.525	70.8±39.4	75.0±36.6	**<0.0001**
SF-36 Bodily pain	80.5±30.8	69.0±23.1	0.963	73.4±28.6	54.7±33.0	0.201	79.1±22.9	81.4±23.1	**0.029**
SF-36 General health	63.6±26.9	61.2±14.9	0.469	59.7±23.9	52.2±18.2	0.390	61.2±21.6	61.5±19.2	**0.005**
SF-36 Vitality	50.0±22.1	52.5±15.7	0.528	56.7±23.5	44.4±13.1	0.685	50.4±14.3	57.0±15.7	**<0.0001**
SF-36 Social functioning	72.9±26.7	77.1±20.0	0.116	75.0±26.4	65.0±36.7	0.770	75.0±21.0	86.2±15.0	**<0.0001**
SF-36 Role emotional	83.3±27.9	77.8±40.4	0.295	59.3±49.4	59.3±46.5	0.672	71.6±36.6	87.7±22.9	**0.001**
SF-36 Mental health	62.7±19.5	66.7±15.3	0.631	64.9±20.3	60.0±21.1	0.969	63.4±15.7	71.9±16.2	**<0.0001**

*Out of N = 1073 patients with at least two visits; **significant p-values (p<0.05) in bold; FDI = Facial Disability Index; FaCE = Facial Clinimetric Evaluation Scale; SF-36 = 36-Item Short Form Survey; NA = not applicable; SD = standard deviation.

Supplement Table S8

Supplement Table S8. Association between patients' / treatment characteristics and probability of recovery after acute facial palsy (N=690*)

Parameter	Categorized	6-month recovery rate %	12-month recovery rate %	log rank p**
All		59.1	67.6	
Gender	Male	61.5	72.8	0.067
	Female	57.1	63.4	
Age, median	<55 years	62.7	69.6	0.717
	≥55 years	55.8	66.7	
Etiology	Idiopathic	72.8	79.5	**<0.0001**
	Traumatic	61.4	68.0	
	Iatrogenic	34.5	48.5	
	Neoplastic	0	0	
	Infectious/Inflammatory	63.8	68.3	
Severity of facial palsy, initial	Incomplete	64.5	72.9	**<0.0001**
	Complete	32.6	41.7	
Stennert index, total score, initial	<median 5	62.6	71.9	**0.001**
	≥median 5	56.5	64.3	
FDI, total score, initial	≤median 75	40.8	50.8	**<0.0001**
	>median 75	70.3	77.3	
FaCE Total score, initial	≤median 77	32.7	43.0	**<0.0001**
	>median 77	72.8	82.6	
SF-36, general health subdomain, initial	<median 62	50.6	60.7	**0.013**
	≥median 62	69.0	81.0	
Stapedius reflex test	Normal	83.5	87.6	**<0.0001**
	Pathological	62.5	66.7	
EMG, PSA	Normal	58.5	67.3	**0.011**
	Pathological	33.1	38.6	
Interval, onset to prednisolone	<median 0	52.3	64.8	NA

	≥median 1	73.2	80.7	
Prednisolone	Yes	64.4	74.0	**<0.0001**
	No	35.0	39.2	
Prednisolone alone	Yes	64.4	70.1	NA
	No	45.0	60.2	
Prednisolone and acyclovir	Yes	74.1	80.9	**<0.0001**
	No	51.1	51.9	

*with at least two visits; **significant p-values (p<0.05) in bold; SI = Stennert index; FDI = Facial Disability index; FaCE= Facial Clinimetric Evaluation Scale; SF-36 = 36-Item Short Form Survey EMG = electromyography; PSA = pathological spontaneous activity.

Supplement Table S9

**Supplement Table S9.** Multivariable Cox regression of prognostic factors for recovery from acute facial palsy (N=690*).					
**Factor**		**HR**	**Lower 95%CI**	**Upper 95%CI**	**p****
**Model 1**					
Initial Stennert index, total score	≥median 5	1	Reference		
	<median 5	1.571	1.026	2.404	**0.038**
Initial FDI, total score	≤median 75	1	Reference		
	>median 75	0.691	0.381	1.251	0.222
Initial FaCE Total score	≤median 77	1	Reference		
	>median 77	2.653	1.519	4.635	**0.001**
Initial SF-36, general health subdomain	<median 62	1	Reference		
	≥median 62	1.232	0.784	1.935	0.365
**Model 2**					
Initial Stennert index, total score	≥median 5	1	Reference		
	<median 5	1.851	1.522	2.250	**<0.0001**
Etiology	Infectious/Inflammatory	1	Reference		
	Idiopathic	1.320	1.000	1.742	**0.050**
	Traumatic	0.798	0.420	1.517	0.491
	Iatrogenic	0.485	0.354	0.665	**<0.0001**
	Neoplastic	0	NA		
**Model 3**					
Initial Stennert index, total score	≥median 5	1	Reference		
	<median 5	1.417	1.051	1.912	0.022
Stapedius reflex test	Pathological	1	Reference		
	Normal	2.077	1.498	2.88	**<0.0001**
EMG, PSA	Pathological	1	Reference		
	Normal	2.329	0.576	9.415	0.236
**Model 4**					
Initial Stennert index, total score	≥median 5	1	Reference		
	<median 5	1.268	0.950	1.691	0.106
Stapedius reflex test	Pathological	1	Reference		
	Normal	2.205	1.610	3.020	**<0.0001**
Prednisolone, alone/combination	No	1	Reference		
	Yes	3.614	2.124	6.149	**<0.0001**
**Model 5**					
Initial Stennert index, total score	≥median 5	1	Reference		
	<median 5	1.315	0.986	1.755	0.062
Stapedius reflex test	Pathological	1	Reference		
	Normal	2.169	1.583	2.972	**<0.0001**
Prednisolone and acyclovir	No	1	Reference		
	Yes	2.230	1.542	3.223	**<0.0001**

*with at least two visits; **significant p-values (p<0.05) in bold; HR = Hazard ratio; CI = confidence interval; SI = Stennert index; FDI = Facial Disability index; FaCE= Facial Clinimetric Evaluation Scale; SF-36 = 36-Item Short Form Survey EMG = electromyography; PSA = pathological spontaneous activity.

SUPPLEMENT FIGURES

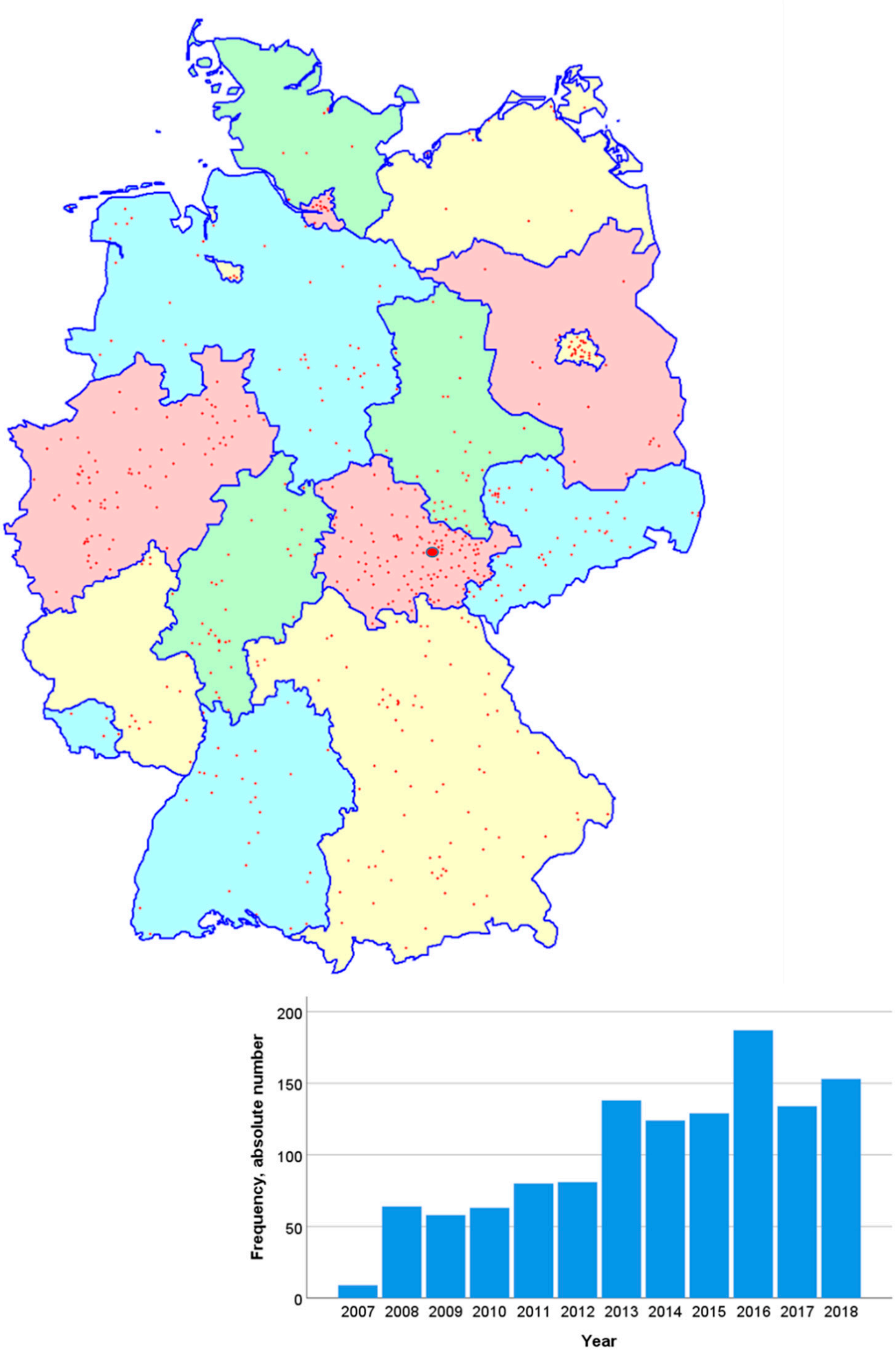

Figure S1. 1212 of the 1220 patients were referred from all over Germany. Eight patients were referred from abroad. The Facial Nerve Center is located in Jena (red spot), a town in the federal state Thuringia. The frequency table in the lower right corner shows a continuously increasing referral from 2007 to 2018.

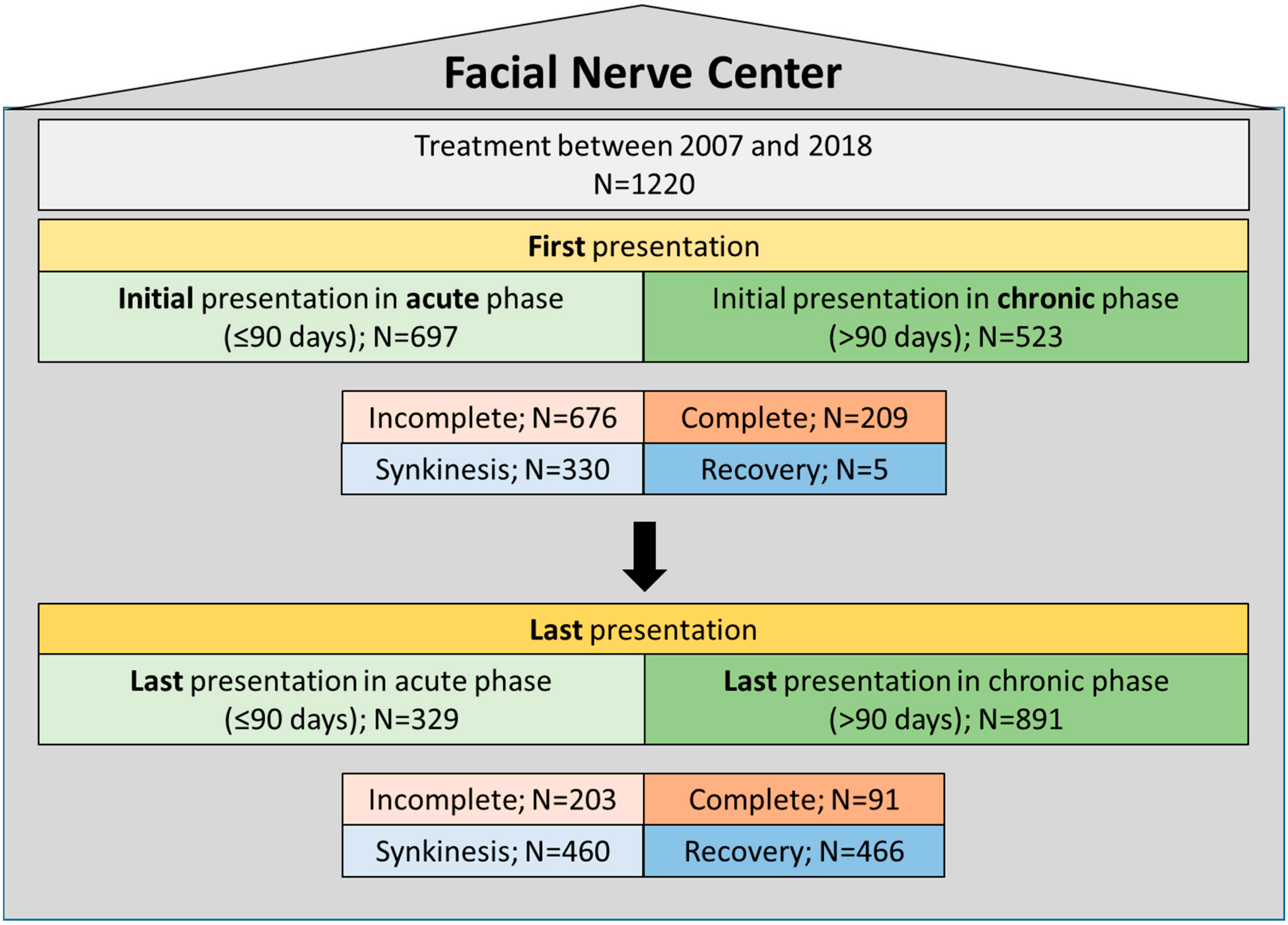

Figure S2. Patients' facial nerve function at first and last presentation in the Facial Nerve center

SUPPLEMENT FIGURE S3

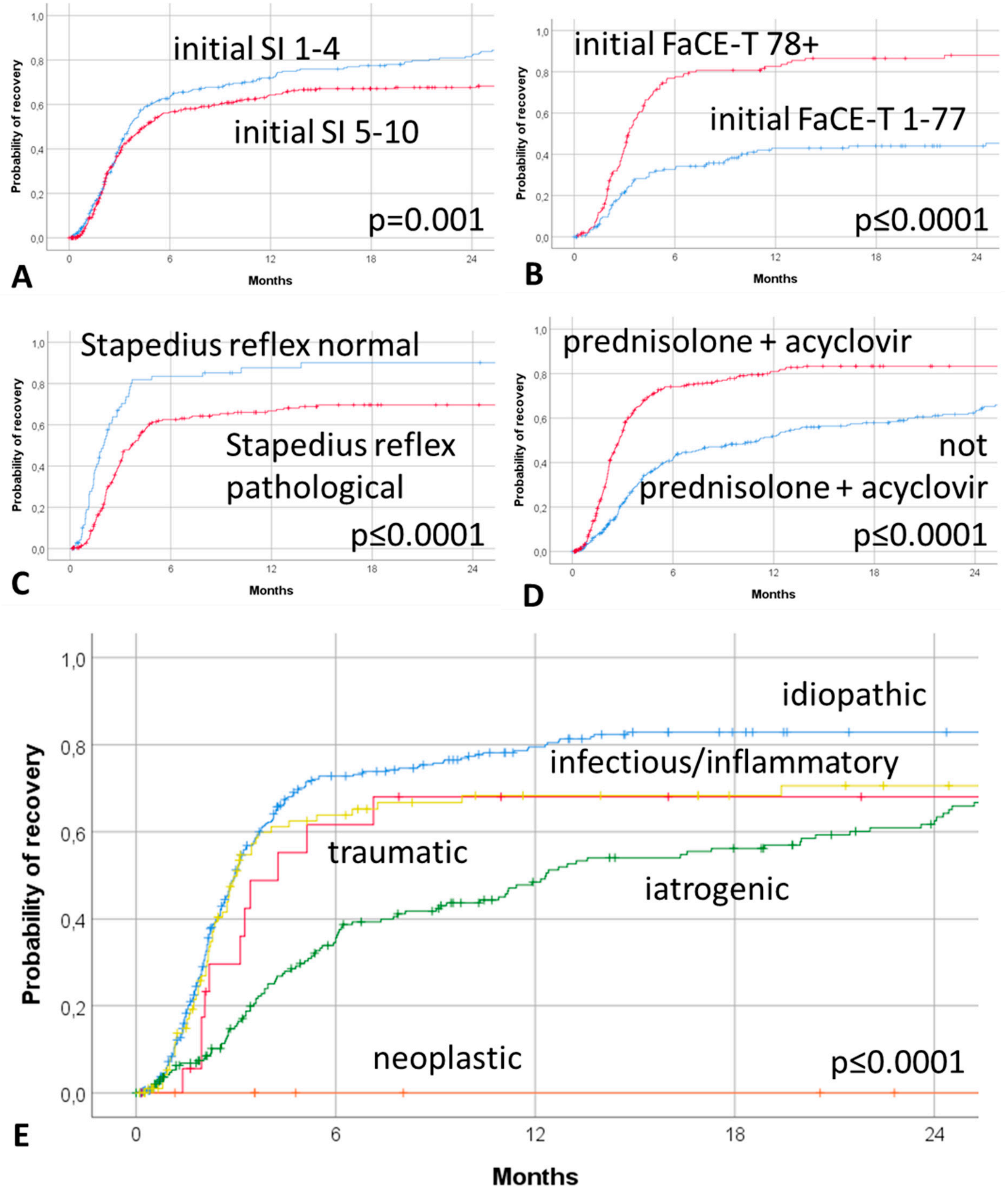

Figure S3. Probability of complete recovery from acute facial palsy related to: A: initial Stennert index (SI); B: initial Facial Clinimetric Evaluation Scale total score (FaCE-T); C: Stapedius reflex test result; D: application of combined prednisolone and acyclovir treatment; E: the etiology. P-values of log-rank tests indicated.

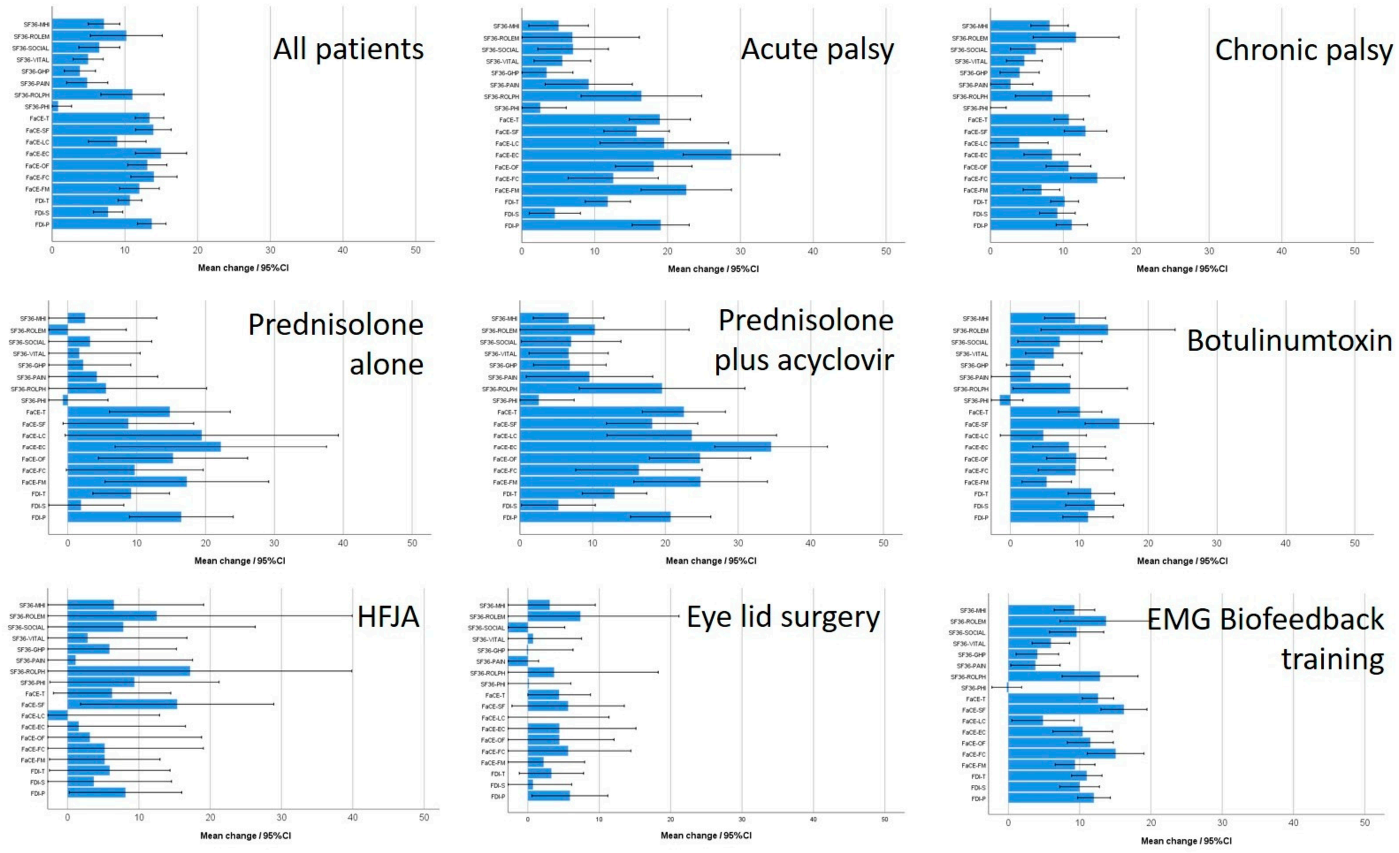

Figure S4. More detailed version of Figure 2: Absolute improvement of facial function between initial and last evaluation in the Facial Nerve Center, assessed with the Facial Disability Index (FDI), the Facial Clinimetric Evaluation Scale (FaCE), and 36-Item Short Form Survey (SF-36) for the entire study group and separately for subgroups of different treatment types.
